# Nondestructive Testing of Pear Based on Fourier Near-Infrared Spectroscopy

**DOI:** 10.3390/foods11081076

**Published:** 2022-04-08

**Authors:** Zhaohui Lu, Ruitao Lu, Yu Chen, Kai Fu, Junxing Song, Linlin Xie, Rui Zhai, Zhigang Wang, Chengquan Yang, Lingfei Xu

**Affiliations:** 1 College of Horticulture, Northwest A&F University, Taicheng Road No. 3, Yangling, Xianyang 712100, China; luzhaohui525@163.com (Z.L.); lrt18508779434@163.com (R.L.); cy1747023889@163.com (Y.C.); junxingsong@163.com (J.S.); zhai.rui@nwafu.edu.cn (R.Z.); wzhg001@163.com (Z.W.); lingfxu2013@sina.com (L.X.); 2 College of Lifescience, Northwest A&F University, Taicheng Road No. 3, Yangling, Xianyang 712100, China; fk2622891245@nwafu.edu.cn; 3 College of Science, Northwest A&F University, Taicheng Road No. 3, Yangling, Xianyang 712100, China; xielinlin0213@126.com

**Keywords:** pear, FT-NIR spectroscopy, quantitative analysis, qualitative analysis

## Abstract

Fourier transform near-infrared (FT-NIR) spectroscopy is a nondestructive, rapid, real-time analysis of technical detection methods with an important reference value for producers and consumers. In this study, the feasibility of using FT-NIR spectroscopy for the rapid quantitative analysis and qualitative analysis of ‘Zaosu’ and ‘Dangshansuli’ pears is explored. The quantitative model was established by partial least squares (PLS) regression combined with cross-validation based on the spectral data of 340 pear fresh fruits and synchronized with the reference values determined by conventional assays. Furthermore, NIR spectroscopy combined with cluster analysis was used to identify varieties of ‘Zaosu’ and ‘Dangshansuli’. As a result, the model developed using FT-NIR spectroscopy gave the best results for the prediction models of soluble solid content (SSC) and titratable acidity (TA) of ‘Dangshansuli’ (residual prediction deviation, RPD: 3.272 and 2.239), which were better than those developed for ‘Zaosu’ SSC and TA modeling (RPD: 1.407 and 1.471). The results also showed that the variety identification of ‘Zaosu’ and ‘Dangshansuli’ could be carried out based on FT-NIR spectroscopy, and the discrimination accuracy was 100%. Overall, FT-NIR spectroscopy is a good tool for rapid and nondestructive analysis of the internal quality and variety identification of fresh pears.

## 1. Introduction

The pear (*Pyrus* spp.) is one of the oldest plants domesticated by humans [1]. The fruits are of high food value, they are tasty, juicy, nutritious and have some health care value. Different types of pears have distinct flavors and textures. China produces more than 60 percent of the world’s pears [2]. Consumers consider the outer quality of the pear, such as size, color and shape, as well as the inner quality of the pear, such as sugar content, acidity and taste. Its post-production treatment, quality evaluation and testing have been important topics in agricultural processing research. The cultivation of pear is increasing in China, and there are large differences between good and bad varieties. Excellent varieties have abundant and stable yields, excellent overall quality and good adaptability, easy to cultivate and are welcomed by the majority of consumers. In contrast, poor quality varieties have low yields, poor quality, poor adaptability and low economic efficiency [3]. At the same time, people’s quality requirements for fresh fruit are becoming more and more demanding, no longer limited to the fruit shape, color and other traditional appearance quality, but more attention to the internal quality indicators of the fruit [4,5,6]. Additionally, the internal quality testing and grading of fresh fruit after harvest has been an important part of fruit commercialization [7]. The soluble solid content (SSC) of fresh fruit influences not only its inherent quality and price, but also its maturity and harvesting duration [7]. Of course, the internal quality of the fruit depends to a large extent on the type and content of organic acids, in addition to the SSC, and different types of organic acids are associated with different taste. Although the organic acid in the ripe pear fruit is mainly malic or citric acid, there are a few varieties of succinic and quinic acid that were detected [8]. Titratable acidity (TA) is frequently used to estimate the ripening time of pears, and as the fruits get closer to ripening, they become less acidic and sweeter in flavor [9]. The ratio of SSC and TA is an important determinant of the flavor quality of pear fruit. These internal parameters are still determined in a destructive manner [10]. Alternatively, traditional quality testing methods and commercial fruit assessment methods are inefficient, require a long time to complete, result in product damage and depend on the estimation of humans. In addition, the pears cannot be sold after being measured and cannot be used for pre-sale grading [11]. Therefore, it is critical to devise a quick and efficient system to determine the quality of pear fruit and identify the cultivar or variety [12].

Near-infrared (NIR) spectroscopy offers the advantages of fast, nondestructive as well as real-time analysis [13]. Useful information is derived from the spectra of multi-molecular absorption bands at different frequencies using Fourier transformed sinusoidal function curves, and the full system of numerous calibration samples with the known composition is represented using a chemometric model. The spectra of these samples are used to calculate calibration functions to serve as models for the analysis of unknown samples [14]. The NIR spectroscopy technique has a great potential for commercialization and practicality as it can determine more than one quality characteristic at the same time, increase the number of samples measured and repeat the analysis of the same samples, and the fruit can still be sold and eaten after testing, without harming the economic efficiency of the producer [15].

Internal quality indicators, such as SSC, hardness, TA, dry matter content and internal disorders, have all been successfully predicted using NIR spectroscopy to assess the quality of fruit [16,17,18,19,20,21,22,23,24,25]. The technology has been applied to determine the cultivars or varieties of soybean seeds (*Glycine max*), rice (*Oryza sativa*), *Dendrobium*, peanuts (*Arachis hypogeae*), wheat (*Triticum aestivum*) and raisins among others [26,27,28,29,30,31,32]. Wu et al. developed a partial least squares regression (PLSR) model to detect SSC content in snow pears using a self-made NIR spectrum detector and an enhanced variable selection approach called the variable stability and cluster analysis algorithm (VSCAA) [9]. Nicolaï et al. compared continuous NIR spectroscopy with time-resolved NIR spectroscopy to predict the SSC in pear [17]. Ying and Liu investigated the use of coupled genetic algorithms for the spectral region selection and quantification of pear SSC and TA using PLS [18]. Paz et al. used PLS to evaluate predictive models that utilized near-infrared spectroscopy to determine the quality of intact pears by assessing SSC and hardness [21]. Sun et al. used visible near-infrared transmission spectroscopy in the 600–904 nm wavelength range to detect brown heart and SSC in pears using a combination of dual wavelength classification and a partial least squares (PLS) quantitative analysis [25]. Han et al. established a discriminant analysis utilizing the Marxian distance model to distinguish normal ‘Yali’ pears from those with brown hearts using transmission spectroscopy in the range of 651–1282 nm [33]. Panmanas Sirisomboon et al. used NIR spectroscopy to determine the content of pectin in Japanese pears in the wavelength range of 1100–2500 nm [34]. Deng and Han used the K-means technique to study cluster analysis and pedigree relationships for various peanuts [30]. Wang et al. used PLS and multiple linear regression (MLR) methods to model the SSC and hardness of the Western Pear (*Pyrus commmunis* L.) and measured the absorption spectra of pears at 500–1010 nm with a visible near-infrared spectrometer [35]. However, there have been few reports of studies that involved detailed analyses that developed a generalized model for multi-factor quality and multiple varieties and variety identification.

**Figure 1:**
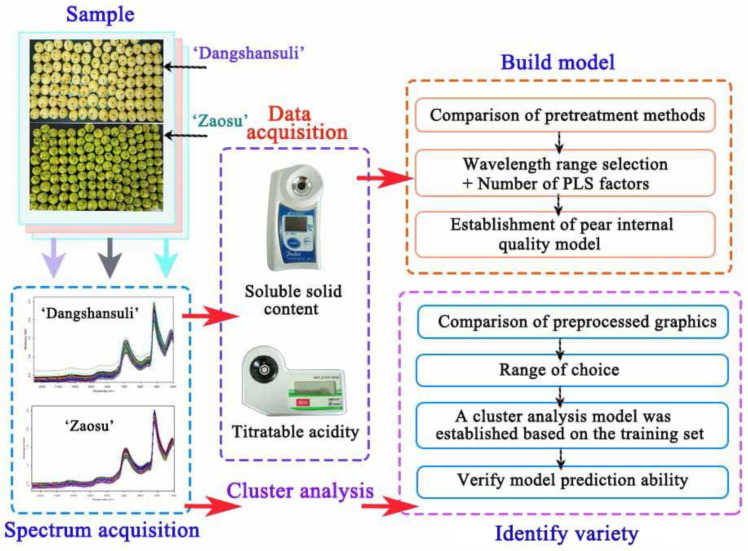
Flow chart of the test and modeling process.

This study addresses the problems of large differences in fruit yield, differences in the quality of fruit after harvesting, better fruit appearance quality but poor edible taste, etc. It focuses on the technical needs of the pear harvesting process which needs to be precise, intelligent and standardized and establishes a pear fruit internal quality nondestructive testing model and variety identification model, with the overall goal of improving the commercial rate and economic efficiency of pear, and provides reference for production practice. Therefore, establishing a universal model for multi-factor quality and the identification of varieties will help the pear industry. The specific objectives were as follows: (1) to investigate the effects of different pretreatments, such as derivatives, multiple scattering correction (MSC) and vector normalization (VN), on the prediction performance; (2) to develop a multi-variety PLS generalized model to predict the SSC and TA of the ‘Zaosu’ and ‘Dangshansuli’ pear cultivars; and (3) to identify ‘Zaosu’ and ‘Dangshansuli’ with NIR using cluster analysis. The spectral distances were calculated based on different methods to compare the accuracy of the established variety models and select the most likely prediction model. These methods obtained the ideal model of NIR spectra to predict pear varieties. The flow chart of the experiment and modeling is shown in Figure 1.

## 2. Materials and Methods

### 2.1. Experimental Samples

**Figure 2:**
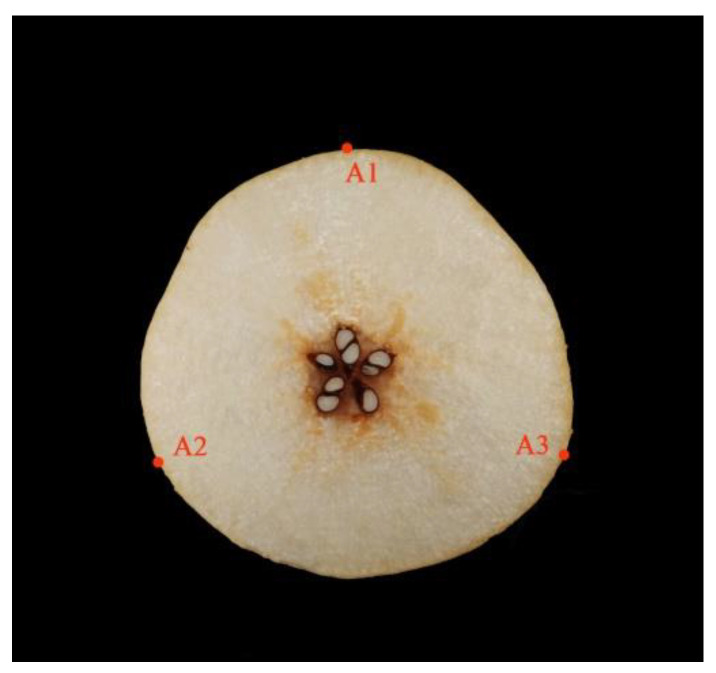
Plot of three measurement points (**A1**, **A2**, **A3**), which are marked around the pear equator and separated by 120°.

Test samples of ‘Zaosu’ (ZS; *P. bretschneideri* Rehd.) and ‘Dangshansuli’ (DS; *P. bretschneideri* Rehd.) were collected on 12 July, 15 July, 20 July, 7 September, 10 September and 25 September 2021, respectively, at Chinese horticultural institutions, such as the Northwest Agriculture and Forestry University (Meixian, Shaanxi province, China), Pucheng pear Experimental Demonstration Station (Pucheng, Shaanxi province, China) and Horticulture Experimental Station of Northwest A&F University (Yangling, Shaanxi province, China), and 340 undamaged pear samples were collected for each variety totaling 680 samples. To avoid the impact of temperature on the test findings, all the pears were transferred to the Northwest Agriculture and Forestry University College of Horticulture (Yangling, China), washed and randomly numbered before being placed in a room at 20 °C and 60% relative humidity for 24 h. Because different locations on the pear fruit contain different information, three equally spaced locations on the equatorial plane of each sample to be measured, as shown in Figure 2 (A1, A2, A3), were chosen to acquire the spectral data and determine the SSC and TA. The spectral data and index measurement value of the samples to be measured were calculated using the average of the measured data at the three points.

### 2.2. Spectral Data Acquisition

The pear samples were scanned by diffuse reflectance spectroscopy using a Fourier transform near-infrared (FT-NIR) spectrometer (MPA; Bruker Optics Ltd., Ettlingen, Germany). The spectrometer was preheated for 40 min before the spectral measurements were acquired. The solid fiber probe was in direct contact with the pear peel, and the spectral data of the pear test site were collected using the OPUS 5.5 software (MPA; Bruker Optics Ltd.). Store in absorbance format, using the internal background as a reference. A chemometrics analysis was performed using OPUS 5.5. A spectral instrument performance test was performed before each test using the self-diagnostic function of the OPUS 5.5 software that was provided with the spectrometer. The parameters were set as follows: the sample was attached with a solid fiber; the measurement range was 12,500–4000 cm^−1^; the instrument resolution was 8 cm^−1^; and 2073 points were scanned.

### 2.3. Quantitative Analysis

#### 2.3.1. SSC and TA Measurement

The SSC and TA of the pears were determined using destructive techniques at the same places as the diffuse reflectance spectral data. First, the juice was obtained at the marker point of the pear, and 1 mL of pear juice was quickly taken up with a pipette drop in the release area of the PAL-1 digital saccharimeter (ATAGO Co., Ltd., Tokyo, Japan) for SSC measurement. Furthermore, 306 μL of pear juice was immediately extracted with a pipette and diluted 100 times with distilled water. A volume of 5 mL of the diluted pear juice was placed on a GMK-835F Pear Acidity Meter (G-won Hitech Co., Ltd., Seoul, Korea) to determine the TA level.

#### 2.3.2. Data Processing and Analysis

The internal quality of fresh pear fruit may be correctly predicted using the right modeling method to develop a regression model. In this study, PLS, one of the most extensively used chemometrics methods, was used to correlate the NIR spectra with the internal quality of pear fruit [36]. The data were preprocessed to reduce bias and changes in distinct linear baselines, as well as to accentuate spectral differences, before being used to develop the PLS regression model [36]. Pre-processing the data ensured a high correlation between the spectral data and content values. Quantitative analysis preprocessing methods include linear compensated difference subtraction (LCS), linear difference subtraction (LDS), vector normalizing (VN), min-max normalization (MMN), multiple scattering correction (MSC), first-order derivatives (FOD) and second-order derivatives (SOD) [37]. The analytical software OPUS 5.5 was used to model the data. The root-mean-square error (RMSE) of the leave-one-out cross-validation is the best factor to construct the calibration model. The leave-one-out cross-validation technique is commonly used to avoid underfitting or overfitting as a result of using latent variables that are too small or large. The smallest RMSE of the cross-validation values determines the ideal number of latent variables [35]. The coefficient of determination (*R*^2^), the corrected mean squared deviation (*RMSECV*) and the residual prediction deviation value (*RPD*) were used as model evaluation indicators. The *R*^2^, the *RMSECV* and the *RPD* are calculated as follows.

$$R^{2}=\left[1-\frac{\sum {\left(d_{i}\right)}^{2}}{\sum {\left(y_{i}-y_{m}\right)}^{2}}\right]\times 100\;RMSECV=\sqrt{\frac{1}{n}\sum {\left(d_{i}\right)}^{2}}\;RPD=\frac{SD}{RMSECV}$$

where *d_i_* is the difference between the *i*th sample’s internal quality index value and the cross-validation determination value; *n* is the number of samples in the statistical calculation; *R* is the number of PLS principal component dimensions; *y_i_* is the analytical value of the internal quality indicator of the ith sample; and *y_m_* is the average value of the internal quality indicators of all the samples. *SD* is the standard deviation of the sample. The *RPD* value is an indicator used to test the robustness of the model. The higher the *RPD* value, the better the model predicts the chemical composition. The model can be used for NIR prediction when the *RPD* is greater than 2.5, and the model has good prediction when it is greater than 3.0 [38]. In this paper, the larger the *R*^2^, the smaller the *RMSECV*, and the model is optimal.

### 2.4. Qualitative Analysis

NIR cluster analysis was used to discriminate between the pear varieties. The cluster analysis pattern recognition method is based on the properties of the samples themselves. Chemometrics use some type of spectral similarity or difference index to determine the affinity between the samples and clusters the samples based on their degree of affinity [39]. These data can be visualized in the form of a tree diagram, diagnostic list or histogram to show the affinity between the spectra. 

The steps of cluster analysis modeling include selecting representative pear fruit samples and measuring their NIR spectra. Various spectral preprocessing methods and different wavebands were examined to mitigate or eliminate the interference of various factors on the spectra, and suitable spectral preprocessing methods were used to preprocess the spectra and select the wavebands to eliminate various spectral interference factors [40]. OPUS 5.5 software has six spectral preprocessing methods to perform qualitative analysis, including FOD, SOD, VN, first-order derivative + vector normalization (FDVN) and second-order derivative + vector normalization (SDVN) where derivatization eliminates baseline shifts [41]. VN allows the measured relative intensity of each spectrum to be consistent with the relative intensity of the true spectrum, and the rotation of the baseline was not eliminated after processing. FDVN basically eliminates the effect of baseline shift and rotation. SDVN is more effective [42]. In this study, the effect of each spectral preprocessing method on the results was examined when modeling to enable the selection of the best preprocessing method. Four methods to calculate the spectral distance were applied in the experiment: standard algorithm (SA), factorial method (FM), first range calibration method (FRC) and reproduction level normalization method (RLN) [43,44]. Select Ward’s algorithm for calculating the distance between the newly created class and all other spectral or classes, which clusters the most homogeneous groups together [45]. From the perspective of variance analysis, it is required that the classification results in the smallest possible intra-class variance and the largest possible inter-class variance [43]. It can be used to generate tree diagrams, bar charts or diagnostic charts to build models. A tree diagram was chosen to display the results of this study because it is more intuitive and easier to use to determine the distance between the categories.

## 3. Results and Discussion

### 3.1. NIR Spectral Features

**Figure 3:**
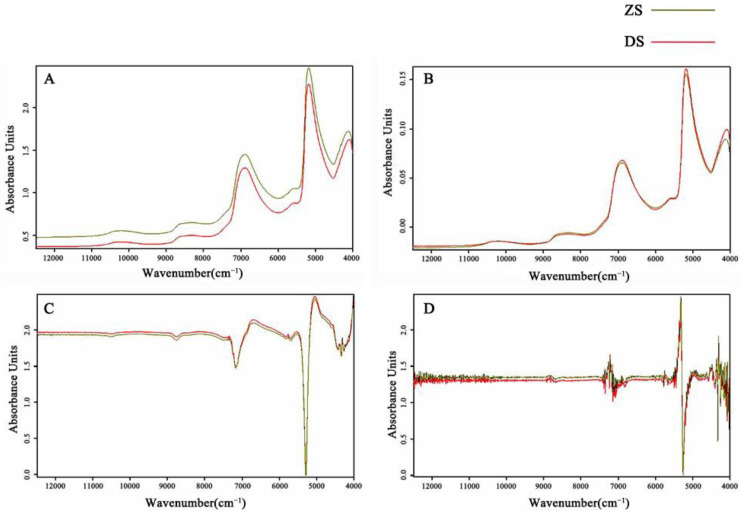
The mean near-infrared spectra (**A**) and the spectra of vector normalizing (**B**), first-order derivative (**C**) and second-order derivative (**D**) of the samples were studied.

Applications of spectroscopic techniques to measure fruit quality are usually performed in the NIR region (4000–12,000 cm^−1^) because spectra in this range contain a wealth of information about O–H, C–H and N–H vibrational absorption [46]. Figure 3A depicts the two spectra that correspond to the two cultivars. Each spectrum is the average of 340 spectra in each cultivar. The diffuse reflectance spectral curve of ZS and DS is exceptionally smooth over the entire NIR spectral region and contains five large absorption peaks. The 5154 cm^−1^ absorption band is related to the water combination band. The O–H first and second overtones of water are associated with the absorption bands at approximately 6900 cm^−1^ and 10,300 cm^−1^, respectively. The C–H first and second overtones are related to bands at 5555–5882 cm^−1^ and 8264–8696 cm^−1^, respectively [47]. 

### 3.2. Quantitative Analytical Model of the FT-NIR

#### 3.2.1. SSC and TA

The maximum, minimum, mean and standard deviation (SD) of 340 ZS and DS fruits were summarized in Table 1. Table 1 shows that the SSC of ZS fruit ranged from 7.37 °Brix to 11.20 °Brix, and the TA ranged from 0.03% to 0.12%. The SSC of DS fruit ranged from 7.37 °Brix to 15.83 °Brix, and the TA ranged from 0.02% to 0.16%. The SSC and TA of tested samples cover a wide enough range to make modeling easier. The acidity of pear fruit consists of a variety of organic acids, and the content of each organic acid will change in different development periods of the fruit, such as malic acid, which shows a trend of increasing and then decreasing during fruit development, while citric acid starts to accumulate in the late stage of fruit development and slightly decreases at maturity, but the total acid content of pear fruit shows a gradual decrease in the process of fruit development. Although the organic acid in the ripe pear fruit is mainly malic acid or citric acid, it still accumulated a certain amount of other components of organic acid and therefore created a different flavor in the pear fruit [48]. For DS and ZS, there was no major difference in total acid content, although the malic acid content in the ripe fruit was relatively large and slightly lower in the latter [8]. The results of our study are consistent with the previous ones, as shown in Table 1.

#### 3.2.2. PLS Modeling of SSC and TA

A multi-species generalized model was developed using PLS to predict the SSC and TA for all the pear samples. PLS is the preferred multivariate correction approach in quantitative research because it can overcome frequent difficulties in this data, such as crosstalk, band overlap and interactions [48]. The smallest RMSE of the cross-validation values determines the ideal number of factors for this model. Internal validation by cross-checking is a widely used method. The performance of the model was assessed by adjusting the RMSECV and R^2^. The spectral data contain useful information about the samples that were tested [49]. However, several types of interference, such as baseline shifts and changes caused by distinct linear baselines, impact the NIR spectra during their measurement. The raw NIR spectral data must be preprocessed before the calibration model can be constructed [50]. To reduce disturbances, mathematical preprocessing methods, such as LCS, LDS, VN, MMN, MSC, FOD and SOD, are commonly utilized. To eliminate or minimize any extraneous spectral information and to increase the chemical information in the spectra, the NIR spectra were treated using several mathematical preprocessing techniques. In addition, to improve the model, the effective wavelength range and the number of PLS components should be determined, and the choice of wavelength range will help to improve the stability of the PLS model [51]. As a result, selecting the best variables is critical for constructing a stable model. In addition, the number of PLS factors is an important issue to consider while calibrating a model since too few variables will result in an underfitted model, while too many factors would degrade the model quality. The optimal conditions for model building were derived by comparing different spectral preprocessing methods, wavelength ranges and number of factors through the automatic “optimization function” of model building, and evaluated by the values of RMSECV, R^2^ and RPD. The larger the R^2^, the higher the RPD value and the smaller the RMSECV, the better the model predicted the chemical composition [5].

In this study, the automatic “optimization function” used for model building was derived from the OPUS 5.5 software [37]. Ye et al. have successfully applied this automatic “optimization function” to the modeling of the volatile compound composition of cider for nondestructive testing. The results showed that the values determined by the reference method based on FT-NIR established for the detection of different volatile compounds correlated well with those determined by the NIR calibration method. The cross-validation and external validation further verified that the FT-NIR-based model for detecting different volatile compounds has good fitting and predictive ability. The model can identify 18 volatile models in cider simultaneously, indicating that the predictive model built using the automatic “optimization function” is excellent and may be applied to the development of more nondestructive testing models [37]. Traditional quality inspection methods have low inspection efficiency, long time required and product destruction, so we need to introduce NIR spectroscopy. In contrast to the Western pear model studied by Wang et al., few studies related to the establishment of a generalized multi-factor quality model for comprehensive analysis on white pear (*P. bretschneideri* Rehd.) systems have been reported [35]. ZS and DS are the main planting varieties of white pear system, and the fruit shape varies greatly, so we initially explore the feasibility of nondestructive testing model inside the white pear.

The optimal settings for the SSC and TA models for ZS and DS, as well as the performance of each calibrated model, are shown in Table 2. It was also demonstrated that using a combination of region selection and data preprocessing to improve the model resulted in lower RMSECV and higher R^2^. The best pretreatment method for SSC and TA is VN. The VN preprocessed spectra are provided in Figure 3B for a more visual examination of the spectra. By choosing an effective wavelength, the variables used in the PLS model are effectively reduced from 2074 to 172–1660.

**Figure 4:**
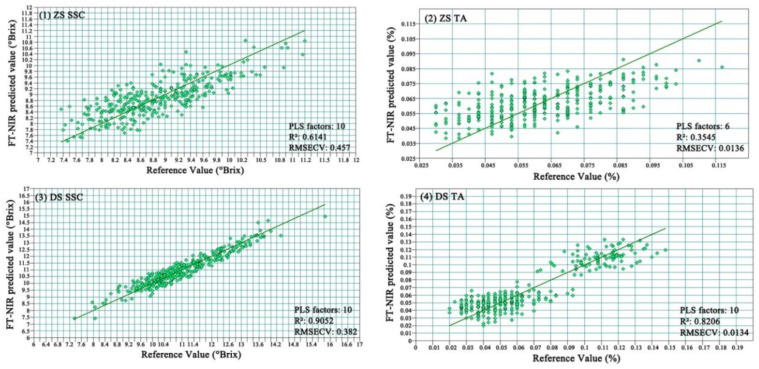
Scatter plot of predicted and measured values of the pear sample prediction model: (**1**) SSC model of ZS; (**2**) TA model of ZS; (**3**) SSC model of DS; (**4**) TA model of DS. PLS, partial least squares; R^2^, coefficient of determination; RMSECV, corrected mean squared deviation.

The cross-test indicated the PLS R^2^, RMSECV and RPD of ZS SSC were 0.6141, 0.526 and 1.407, respectively. The R^2^, RMSECV and RPD for ZS TA were 0.3545, 0.0136 and 1.471, respectively. The R^2^, RMSECV and RPD of DS SSC were 0.9052, 0.382 and 3.272, respectively. The R^2^, RMSECV and RPD of DS TA were 0.8206, 0.0134 and 2.239, respectively, as shown in Figure 4. The SSC and TA models predicted better for DS compared with ZS, and the cross-validation tentatively proved that the models were feasible. Further investigation would be necessary for validation and optimization of the accuracy of the model for DS.

### 3.3. Qualitative Analytical Model of the FT-NIR

#### 3.3.1. Determination of the Model Parameters

The clustering analysis method in the OPUS 5.5 software was used to classify the similar spectra by groups to identify and distinguish ZS and DS. The preprocessing methods in the cluster analysis included VN, FOD and SOD and a combination of both. As shown in Figure 3B, the vector-normalized spectrograms of the two species were highly similar. Although the FOD enhanced the steepness of several absorption peaks, the spectra of the two species have a striking resemblance and were difficult to interpret as shown in Figure 3C. Figure 3D shows that after the SOD treatment, the absorbance of the two pears was highly variable at the same wavelength range of 7500–4000 cm^−1^, and the amount of information available for the study was richer. Furthermore, the main absorption region in the NIR is the octave and combined frequency region that contains hydrogen at all levels. Overall, 4000–5000 cm^−1^ is the combined frequency region, 5000–9000 cm^−1^ is the first octave region and 9000–12,000 cm^−1^ is the second octave region where the second octave region has serious spectral drift, weak intensity, end effect and the system error will also result in large noise at the end of the spectral curve. Therefore, the secondary octave region is generally not used as the area for analysis. Owing to the presence of noise in the tail, the spectral range of 7500–5000 cm^−1^ was chosen for the study after comprehensive consideration.

**Figure 5:**
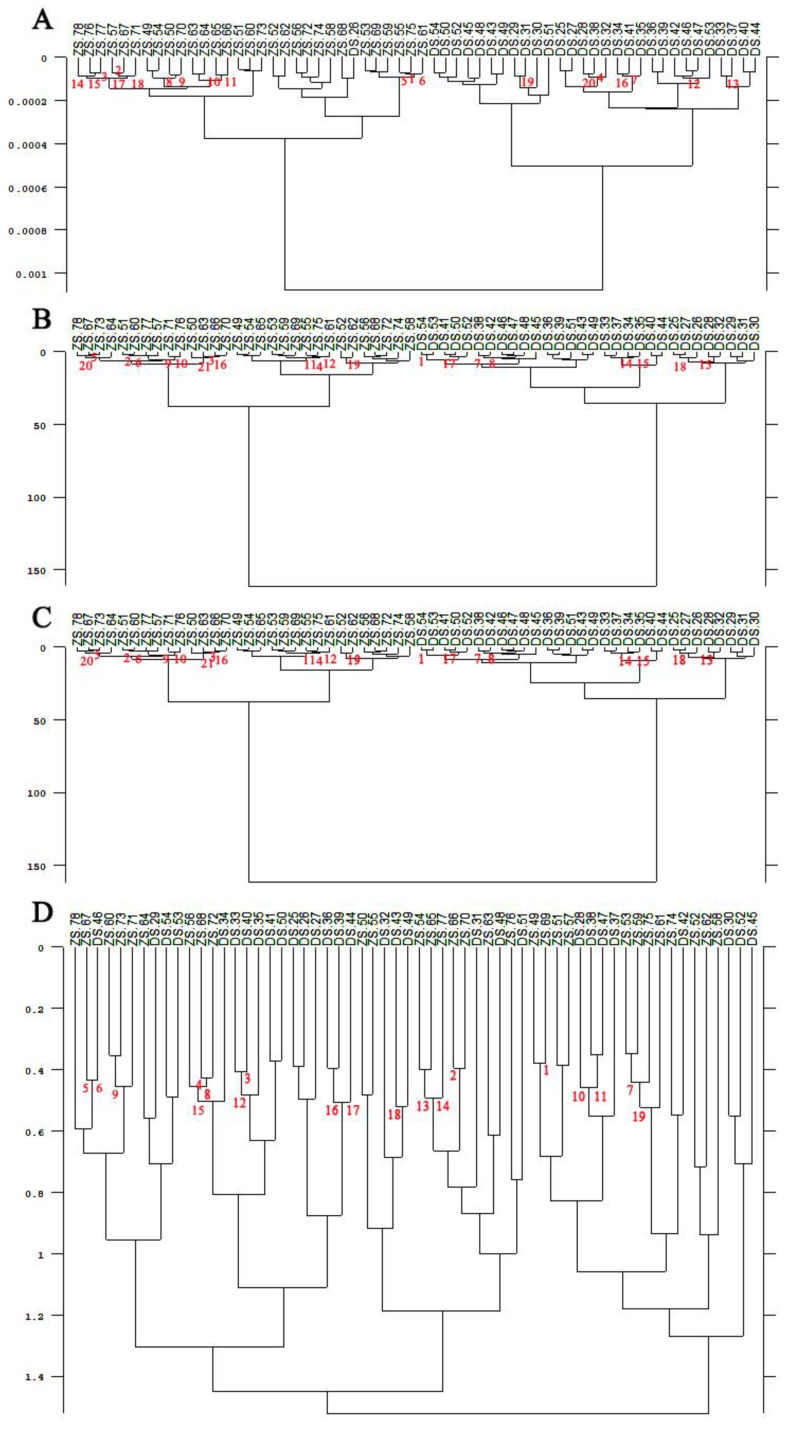
Tree diagram of pear fruit clustering analysis from the Meixian test site. (**A**) Standard algorithms; (**B**) First range calibration methods; (**C**) Reproduction level normalization methods; (**D**) Factorial methods.

The three batches of ZS and DS collected in Section 2.1 were constructed into three cluster analytical models, and 50 pears from each batch of different varieties were selected as samples. The first 30 were used as the training set and the last 20 as the prediction set. Three hundred fruit were used in total (150 each for ZS and DS). As shown in Table 3, the spectra were added to the list, the spectral range was selected, the preprocessing method was SOD and nine smoothing points were used. Four different methods for calculating spectral distances were used separately throughout the experiment to compare the accuracy of variety identification. Additionally, Ward’s algorithm method was selected for cluster analysis to produce a tree diagram, as shown in Figure 5.

**Figure 6:**
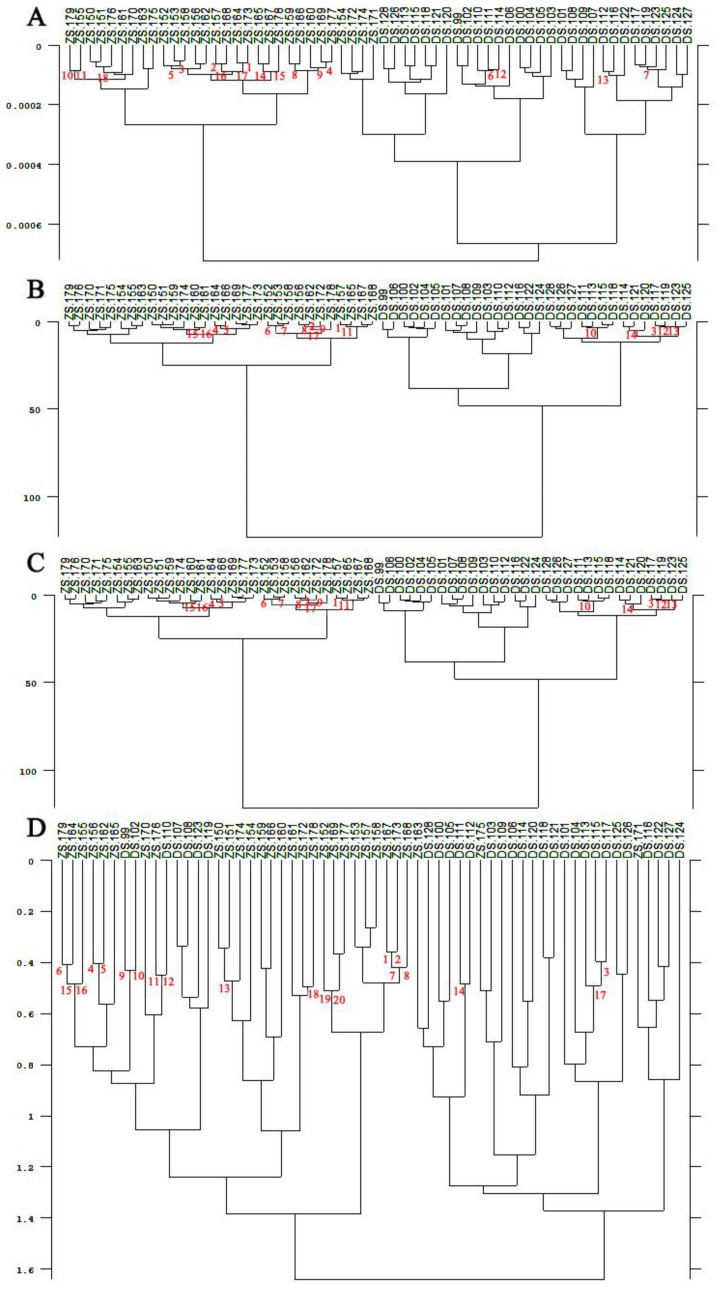
Tree shape of pear fruit clustering analysis from the Pucheng Pear Experimental Demonstration Station. (**A**) Standard algorithms; (**B**) First range calibration methods; (**C**) Reproduction level normalization methods; (**D**) Factorial methods.

**Figure 7:**
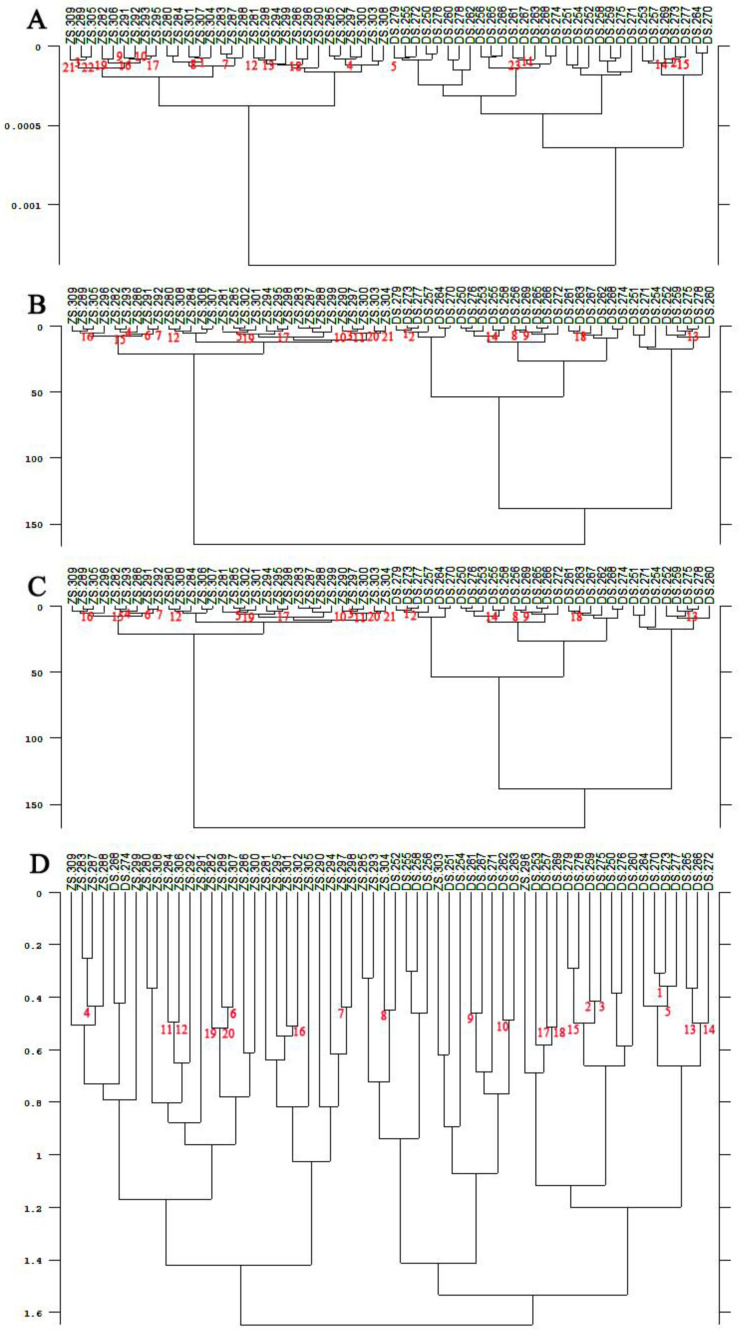
Cluster analysis tree diagram of pear fruit from the Horticulture Experimental Station of Northwest A&F University. (**A**) Standard algorithms; (**B**) First range calibration methods; (**C**) Reproduction level normalization methods; (**D**) Factorial methods.

According to the clustering analysis, it can be seen from Figure 5 and Figure 6 that ZS and DS picked at the Meixian test site and Pucheng Pear Experimental Demonstration Station could not be effectively clustered into two classes using the SA and FM. Whereas the FRC and the RLN could clearly cluster the pear fruits of ZS and DS varieties into two categories. From Figure 7, it can be seen that the pear fruits picked at the Horticulture Experimental Station of Northwest Agriculture and Forestry University can be clustered into two categories by the SA, the FRC and the RLN for both ZS and DS. Additionally, the FM will produce misclassification of the two pear fruits. In summary, among the four different methods of calculating spectral distances, the use of the FRC and the RLN do not cause misclassification of ZS and DS. It can be used to discriminate the varieties of pear fruit NIR spectra in different locations and at different times, indicating that the specificity of the model is good.

#### 3.3.2. Validation of the Predictive Capability of Model

To verify the accuracy and predictive ability of the model, 20 samples of different species and different batches were selected as the prediction set. After the model was well established, the prediction was conducted by entering the cluster analysis test interface, storing it with the FRC and accessing the already stored cluster analysis method file. The same preprocessing method and range selection were used for the prediction set samples and the training set samples. The level of cluster analysis indicated the variability among cultivars to some extent. It was highly effective at discriminating between the cultivars if the cultivar characteristics were highly accurate. However, a variety accuracy of 0 indicated that there was no difference between the varieties, and the characteristic cannot discriminate between the samples. The codes to establish the results of individual clustering tests are shown in Table 4 to make it easier to evaluate the findings. All three discriminant analysis models have a clustering result of 1 and an accuracy of 100%.

## 4. Conclusions

The modeling of SSC and TA in pear fresh fruits and variety identification were conducted in this study using a combination of near-infrared spectroscopy and chemometrics, with the research objects being ZS and DS. The predictive model for pear fruit was constructed using the PLS method in combination with various spectral preprocessing methods, which can remove noise and offset the baseline and bias, to eliminate or minimize any unnecessary spectral information and enhance the chemical information in the spectra, effectively improving the stability and validity of the model. It was shown that the PLS method was more effective at predicting the SSC and TA of DS compared with ZS. The applicability of the cluster analysis model can be used for pear fruit varieties in different locations and at different times, indicating that the model is highly specific and providing a new method to identify pear varieties. In future work, research should validate and optimize the DS model to make it more accurate, possibly continue to validate in the industry in order to generalize and further study the multi-species modeling of fruits, especially to improve the performance of the ZS model.It provides technical guidance for pear internal quality nondestructive testing and a classification system to promote the development of the world pear industry.

## Figures and Tables

**Table 1:** Soluble solid content (SSC) and titratable acidity (TA) of ‘Zaosu’ and ‘Dangshansuli’ pear cultivars.

Variety	SSC (°Brix)				TA (%)			
	Max	Min	Average	SD	Max	Min	Average	SD
ZS	11.20	7.37	8.88	0.74	0.12	0.03	0.06	0.02
DS	15.83	7.37	10.99	1.25	0.16	0.02	0.07	0.03

Max, maximum; Min, minimum; SD, standard deviation; SSC, soluble solids content; TA, titratable acidity.

**Table 2:** Performance parameters of a pear variety calibration model with optimized data preprocessing methods.

Parameters	Pretreatment Method	Effective Wavenumber Range (cm^−1^)	PLS Factors	Variables	Cross-Validation		
					R^2^	RMSECV	RPD
ZS SSC	VN	12,493.2–6098.1	10	1660	0.6141	0.526	1.407
ZS TA	VN	6102–5446.3	6	172	0.3545	0.0136	1.471
DS SSC	VN	12,493.2–6098.1	10	1660	0.9052	0.382	3.272
DS TA	VN	6102–5446.3	10	172	0.8206	0.0134	2.239

PLS, partial least squares; SSC, soluble solid content; TA, titratable acidity; VN, vector normalization; R^2^, coefficient of determination; RMSECV, corrected mean squared deviation; RPD, residual prediction deviation.

**Table 3:** Sample selection information.

Collection Location	Sample Set	Number of Samples	Training Set	Prediction Set
Meixian test site	ZS	50	30	20
	DS	50	30	20
Pucheng Pear Experimental Demonstration Station	ZS	50	30	20
	DS	50	30	20
Horticulture Experimental Station of Northwest A&F University	ZS	50	30	20
	DS	50	30	20

**Table 4:** Clustering test result codes.

Clustering Results	Code
Test results OK	1
No clustering test was performed	0
Test result error	−1

## Data Availability

No new data were created or analyzed in this study. Data sharing is not applicable to this article.
